# The Texas Anti-Tubereulosis Commission

**Published:** 1911-08

**Authors:** 


### The Texas Anti-Tuberculosis Commission.

The Texas State Medical Journal, in its latest number, speaks, not in anger, but in sorrow, of the manner in which Governor Colquitt has composed the anti-tuberculosis commission. The law provides that the commission shall be composed of five members, two of whom shall be the Governor himself and the State Health Officer. The three others are referred to in the law as "citizens." The Governor is a citizen, the State Health Officer is a citizen, and so are most of the doctors of the State, lawyers, icemen, dentists, blacksmiths and perhaps 95 per cent of the men in every other avocation and vocation. But evidently Governor Colquitt found it convenient to construe "citizen" to mean almost any one who is not a doctor, and none of these three places, each affording a salary of $1800 a year and traveling expenses, has gone to a doctor.

The law provides that "the three citizens on said commission shall devote their entire time to the work of caring for said institutions and of lecturing to and educating the people of Texas upon the treatment and prevention of tuberculosis."

We do not recall at the moment the names of the gentlemen whom Governor Colquitt appointed, nor is the question of their personalities of enough moment to warrant a search through the files for their names. The point of the matter is that it must seem highly improbable that three men who are not physicians are qualified to satisfy that provision of the law which requires them to educate the people "on the treatment and prevention of tuberculosis." If the lectures which these commissioners are required to give are to have their intended effect, we should think it would be necessary, or at least highly desirable, that they should be delivered by men who understand the subject much better than the laymen can understand it. It may be, of course, that they can make themselves highly proficient, but the purpose of the law was, not to pay for the education of three men in this subject, but to bring about the diffusion of knowledge which three men already have of it.

Possibly Governor Colquitt's interpretation of the law would be affirmed by any skilled constructionist of legal terminology, but if so, that fact only reproaches Governor Colquitt for not having vetoed a measure which provides places for men who, in the ordinary run of events, could not be capable of performing the duties efficiently. It might be that one member might well be a layman and on him imposed the burden of "caring for said institutions," but the spectacle of a semi-professional man "lecturing to and educating the people of Texas upon the treatment and prevention of tuberculosis" is one that is apt to excite their risibilities rather than their fears as to this dread scourge.

Speaking postscriptively, how many lectures have these three gentlemen delivered since their appointment, some two or more months ago? Or, is it that they have not yet finished "cramming" on the subject preparatory to their several tours of enlightenment? —*Dallas News,* July 12, 1911.

